# Hypoglycemia risk with inappropriate dosing of glucose-lowering drugs in patients with chronic kidney disease: a retrospective cohort study

**DOI:** 10.1038/s41598-023-33542-z

**Published:** 2023-04-19

**Authors:** Yun-Jhe Li, Yuh-Lih Chang, Yueh-Ching Chou, Chia-Chen Hsu

**Affiliations:** 1 Department of Pharmacy, Taipei Veterans General Hospital, No. 201, Sect. 2, Shih-Pai Road, Taipei, 112 Taiwan; 2 Department of Pharmacy, Shuang Ho Hospital, Taipei Medical University, New Taipei, Taiwan; 3 Institute of Pharmacology, College of Medicine, National Yang Ming Chiao Tung University, Taipei, Taiwan; 4 Department of Pharmacy, National Yang Ming Chiao Tung University, Taipei, Taiwan

**Keywords:** Endocrinology, Medical research, Nephrology

## Abstract

The incidence rates and consequences of inappropriate dosing of glucose-lowering drugs remain limited in patients with chronic kidney disease (CKD). A retrospective cohort study was conducted to estimate the frequency of inappropriate dosing of glucose-lowering drugs and to evaluate the subsequent risk of hypoglycemia in outpatients with an estimated glomerular filtration rate (eGFR) of < 50 mL/min/1.73 m^2^. Outpatient visits were divided according to whether the prescription of glucose-lowering drugs included dose adjustment according to eGFR or not. A total of 89,628 outpatient visits were included, 29.3% of which received inappropriate dosing. The incidence rates of the composite of all hypoglycemia were 76.71 and 48.51 events per 10,000 person-months in the inappropriate dosing group and in appropriate dosing group, respectively. After multivariate adjustment, inappropriate dosing was found to lead to an increased risk of composite of all hypoglycemia (hazard ratio 1.52, 95% confidence interval 1.34, 1.73). In the subgroup analysis, there were no significant changes in the risk of hypoglycemia regardless of renal function (eGFR < 30 vs. 30–50 mL/min/1.73 m^2^). In conclusion, inappropriate dosing of glucose-lowering drugs in patients with CKD is common and associated with a higher risk of hypoglycemia.

## Introduction

Chronic kidney disease (CKD) is a major public health problem worldwide. In 2017, the global prevalence of CKD was 9.1%, which is approximately 700 million people, and 1.23 million people died of CKD^1,2^. The disease imposes a significant physical, psychological, and economic burden on patients, their families, and health care systems^3–5^.

In clinical practice, many drugs require dose adjustment according to patient renal function. Among patients with renal insufficiency, inappropriate doses are common^6–11^ and can result in patient harm^12–14^. A systematic review showed that glucose-lowering drugs were most associated with inappropriate use in nonhospitalized patients with renal insufficiency^10^. Previous studies have reported that in patients with CKD, approximately 30% of metformin and up to 40% of dipeptidyl peptidase-4 (DPP-4) inhibitors were not dosed according to the patient’s renal function^13,15,16^. Inappropriate renal dosing of DPP-4 inhibitors significantly increases the risk of mortality, emergency department visits, and severe hypoglycemia^13^.

However, with a variety of glucose-lowering drugs becoming available, the amount of real-world evidence regarding the incidence rates and consequences of inappropriate dosing of these medications remains limited in patients with CKD. Impaired renal function may have a critical effect on the pharmacokinetics of most glucose-lowering drugs, thus exposing patients to a higher risk of side effects, primarily hypoglycemic episodes^17^. This study aimed to estimate the frequency of inappropriate dosing of glucose-lowering drugs and evaluate the subsequent risk of hypoglycemia in patients with CKD.

## Methods

### Ethics approval

This study was approved by the Institutional Review Board of Taipei Veterans General Hospital (TPEVGH IRB; No. 2020-10-004BC) and conducted according to the principles of the Declaration of Helsinki. Because the investigation posed minimal risk to the participants and did not involve procedures, the requirement for written informed consent from the patients was waived by the TPEVGH IRB.

### Setting

This retrospective cohort study was conducted at an academic medical center (TPEVGH), which provides more than 2.5 million outpatient visits for 1.1 million outpatients each year in northern Taiwan. On average, approximately 25,000 drug prescriptions are generated daily for ambulatory patients. We retrieved the data for this study from electronic medical records provided by the Big Data Center, TPEVGH. The interpretations and conclusions contained here do not represent the position of TPEVGH.

### Study design and data collection

We included outpatients who were prescribed target drugs between January 1, 2015, and December 31, 2018, and who had an estimated glomerular filtration rate (eGFR) < 50 mL/min/1.73 m^2^. Patients younger than 20 years were excluded because they were vulnerable subjects, as specified by the TPEVGH IRB.

The target drugs used this study were glucose-lowering drugs requiring dose adjustments based on the patient renal function and that were available in the study hospital, including the following 10 medications: acarbose, alogliptin, dapagliflozin, empagliflozin, gliclazide, glimepiride, metformin, saxagliptin, sitagliptin, and vildagliptin (Supplementary Table 1). We calculated the surrogate marker of renal function, eGFR, using the body surface area modified Cockcroft–Gault formula^18,19^. Laboratory data were collected from the day of the visit or the nearest day before the visit.

We defined inappropriate prescriptions for renal dosing as a dose of the target drug exceeding the dosing recommendations based on renal function or a condition in which the target drug is not recommended or contraindicated, as shown in Supplementary Table 1. The dosing recommendations provided by the 2018 drug package labels in Taiwan.

Exposure was defined as an inappropriate dosing of glucose-lowering drugs. If any inappropriate prescription was ordered at the time of the outpatient visit, that visit was categorized into the inappropriate dosing group. If no inappropriate prescription was ordered, that visit was categorized into the appropriate dosing group. We compared the risk of hypoglycemia between the two groups. The cohort entry day was defined as the date of each outpatient visit for the patients. We followed up patients from the cohort entry day to the earliest occurrence of hypoglycemia, the date of the next outpatient visit during which the patient received target medications, loss of follow-up, or December 31, 2018. Loss of follow-up was defined as a patient who did not return to our outpatient clinic for follow-up and who was receiving target medications after the prescription days plus the grace period, which was defined as half of the prescription days^20^. Supplementary Fig. 1 shows the potential patterns of follow-up.

### Outcomes measurement

The outcomes were hypoglycemic events, including the composite of all hypoglycemia, severe hypoglycemia, moderate hypoglycemia, and mild hypoglycemia. Severe hypoglycemia was defined as an emergency department visit due to hypoglycemia (blood glucose level < 70 mg/dL or *International Classification of Disease, Clinical Modification* codes, as shown in Supplementary Table 2)^21–23^. Moderate hypoglycemia was defined as an outpatient visit with a blood glucose level < 54 mg/dL, and mild hypoglycemia was defined as an outpatient visit with blood glucose level between 54 and 69 mg/dL. The composite of all hypoglycemia was defined as the occurrence of any of the above hypoglycemic events.

### Baseline covariates

The baseline period comprised the cohort entry date to 1 year before the cohort entry date. We identified potential confounders, including age, sex, eGFR, HbA_1C_, Charlson Comorbidity Index^24,25^, number of previous severe hypoglycemic events, number of concurrent use of glucose-lowering drugs, sulfonylurea use, and insulin use. We retrieved the current state at the cohort entry date for age, sex, number of concurrent use of glucose-lowering drugs, sulfonylurea use, and insulin use. For eGFR and HbA_1C_, we retrieved the data closest to the cohort entry date. For the number of previous severe hypoglycemic events and Charlson Comorbidity Index, we identified these two covariates throughout the baseline period.

### Statistical analysis

We analyzed the baseline characteristics using descriptive statistics. Continuous variables were described as mean ± standard deviation (SD) and categorical variables as frequencies and percentages. We calculated the standardized differences to assess the covariate balances between groups. Standardized differences greater than 0.1 or less than − 0.1 were considered significant. The incidence rate of hypoglycemia was calculated as events per 10,000 person-month. We used the Kaplan–Meier method to estimate the cumulative incidence curve and also conducted the log-rank test. A marginal Cox proportional hazard model was used, to take into account a possible clustering effect of multiple prescriptions per patient^26^. This model takes into account the censored nature of the data and possible intra-cluster dependence using a robust sandwich covariate matrix estimate. Hazard ratios (HRs) and 95% confidence intervals (CIs) were reported. *P* < 0.05 was considered significant.

We conducted a subgroup analysis stratified by eGFR to assess whether poorer renal function could influence the effects of inappropriate dosing of glucose-lowering drugs on the risk of hypoglycemia. We stratified the patients into two groups based on their renal function: eGFR 30–50 mL/min/1.73 m^2^ and eGFR < 30 mL/min/1.73 m^2^. To test for robustness of our study findings, we performed sensitivity analyzes, restricting the inclusion of the study to outpatient visits without insulin prescriptions, patients without dialysis, and using the CKD Epidemiology Collaboration (CKD-EPI) equation to calculate GFR^27^.

We performed all analyses using SAS software version 9.4 (SAS Institute Inc, Cary, NC), STATA 12.1 (Stata Corp. Statistical Software), and R software version 4.0.3 (The R Foundation for Statistical Computing).

## Results

A total of 89,628 outpatient visits were included, of which metformin, glimepiride, and acarbose were the most frequently prescribed drugs. The frequency of drug prescription according to the patient’s renal function is shown in Supplementary Table 3.

### Characteristics of inappropriate and appropriate dosing groups

Of all the visits, 26,268 (29.3%) were in the inappropriate dosing group. Table 1 shows the baseline characteristics of the two groups. Patients in the inappropriate dosing group were older (age mean ± SD: 80.8 ± 10.1 vs. 79.2 ± 9.9 years), had a lower eGFR (mean ± SD: 29.8 ± 10.3 vs. 39.6 ± 8.2 mL/min/1.73 m^2^), and had a higher number of glucose-lowering drugs prescribed at that visit (mean ± SD: 2.0 ± 0.9 vs. 1.7 ± 0.8). Patients in the inappropriate dosing group were more likely to receive sitagliptin (33.6% vs. 11.7%), saxagliptin (16.7% vs. 0.9%), and gliclazide (18.5% vs. 12.8%) and less likely to receive metformin (43.8% vs. 65.2%), linagliptin (15.0% vs. 20.0%), and empagliflozin (0.5% vs. 3.0%) than those in the appropriate dosing group.

**Table 1:** Baseline characteristics of patients in the inappropriate dosing and appropriate dosing groups.

Variable	Inappropriate dosing group, n = 26,268 (100%)	Appropriate dosing group, n = 63,360 (100%)	Absolute standardized difference
Patient factors			
Age, y, mean ± SD	80.8 ± 10.1	79.2 ± 9.9	0.16
Male, n (%)	16,145 (61.5)	38,614 (60.9)	0.01
eGFR, mL/min/1.73 m^2^, mean ± SD	29.8 ± 10.3	39.6 ± 8.2	1.06
HbA_1C_, %, mean ± SD	7.4 ± 1.4	7.3 ± 1.3	0.05
History of severe hypoglycemia, n (%)			0.02
0	26,051 (99.2)	62,925 (99.3)	
≥ 1	217 (0.8)	435 (0.7)	
Comorbidities			
CCI, mean ± SD	3.1 ± 1.8	2.9 ± 1.8	0.07
Diabetes with chronic complications, n (%)	8,567 (32.6)	18,209 (28.7)	0.08
Cerebrovascular disease, n (%)	2,049 (7.8)	4,764 (7.5)	0.01
Any malignancy, n (%)	1,675 (6.4)	5,321 (8.4)	0.08
Congestive heart failure, n (%)	1,361 (5.2)	2,699 (4.3)	0.04
Dementia, n (%)	837 (3.2)	1,922 (3.0)	0.01
Chronic pulmonary disease, n (%)	682 (2.6)	2,105 (3.3)	0.04
Peptic ulcer disease, n (%)	558 (2.1)	1,423 (2.3)	0.01
Mild liver disease, n (%)	486 (1.9)	1,847 (2.9)	0.07
Hemodialysis, n (%)	475 (1.8)	620 (1.0)	0.07
Myocardial infarction, n (%)	277 (1.1)	637 (1.0)	0.01
Connective tissue disease, n (%)	159 (0.6)	362 (0.6)	0.00
Anemia, n (%)	122 (0.5)	168 (0.3)	0.03
Peripheral vascular disease, n (%)	110 (0.4)	229 (0.4)	0.00
Metastatic solid tumor, n (%)	47 (0.2)	91 (0.1)	0.03
Moderate or severe liver disease, n (%)	10 (0.0)	30 (0.1)	0.04
Paraplegia and hemiplegia, n (%)	8 (0.0)	17 (0.0)	0.00
Concurrent use of glucose-lowering drugs			
No. of glucose-lowering drugs, mean ± SD	2.0 ± 0.9	1.7 ± 0.8	0.34
Metformin, n (%)	11,502 (43.8)	41,294 (65.2)	0.44
DPP-4 inhibitors			
Sitagliptin, n (%)	8,812 (33.6)	7,406 (11.7)	0.54
Saxagliptin, n (%)	4,379 (16.7)	590 (0.9)	0.58
Linagliptin, n (%)	3,937 (15.0)	12,640 (20.0)	0.13
Vildagliptin, n (%)	2,654 (10.1)	6,586 (10.4)	0.01
Alogliptin, n (%)	7 (0.0)	92 (0.2)	0.04
Sulfonylureas			
Glimepiride, n (%)	7,334 (27.9)	15,798 (24.9)	0.07
Gliclazide, n (%)	4,871 (18.5)	8,115 (12.8)	0.16
Acarbose, n (%)	6,195 (23.6)	12,471 (19.7)	0.09
Insulins, n (%)	4,490 (17.1)	11,127 (17.6)	0.01
Short-acting, n (%)	307 (1.2)	998 (1.6)	0.03
Intermediate-acting, n (%)	585 (2.2)	1,151 (1.8)	0.03
Long-acting, n (%)	2,709 (10.3)	6,593 (10.4)	0.00
Premixed, n (%)	1,183 (4.5)	3,413 (5.4)	0.04
SGLT-2 inhibitors			
Empagliflozin, n (%)	134 (0.5)	1,876 (3.0)	0.19
Dapagliflozin, n (%)	137 (0.5)	88 (0.1)	0.07
Meglitinides			
Repaglinide, n (%)	923 (3.5)	1,568 (2.5)	0.06
Mitiglinide, n (%)	616 (2.4)	1,161 (1.8)	0.04
Nateglinide, n (%)	130 (0.5)	219 (0.4)	0.02
Pioglitazone, n (%)	88 (0.3)	200 (0.3)	0.00
GLP-1 analogues			
Liraglutide, n (%)	7 (0.0)	128 (0.2)	0.05
Dulaglutide, n (%)	2 (0.0)	25 (0.0)	0.02

CCI, Charlson Comorbidity Index; DPP-4 inhibitor, dipeptidyl peptidase-4 inhibitor; SGLT-2 inhibitor, sodium-glucose cotransporter 2 inhibitor.

### Associations between inappropriate dosing and hypoglycemia

**Figure 1:**
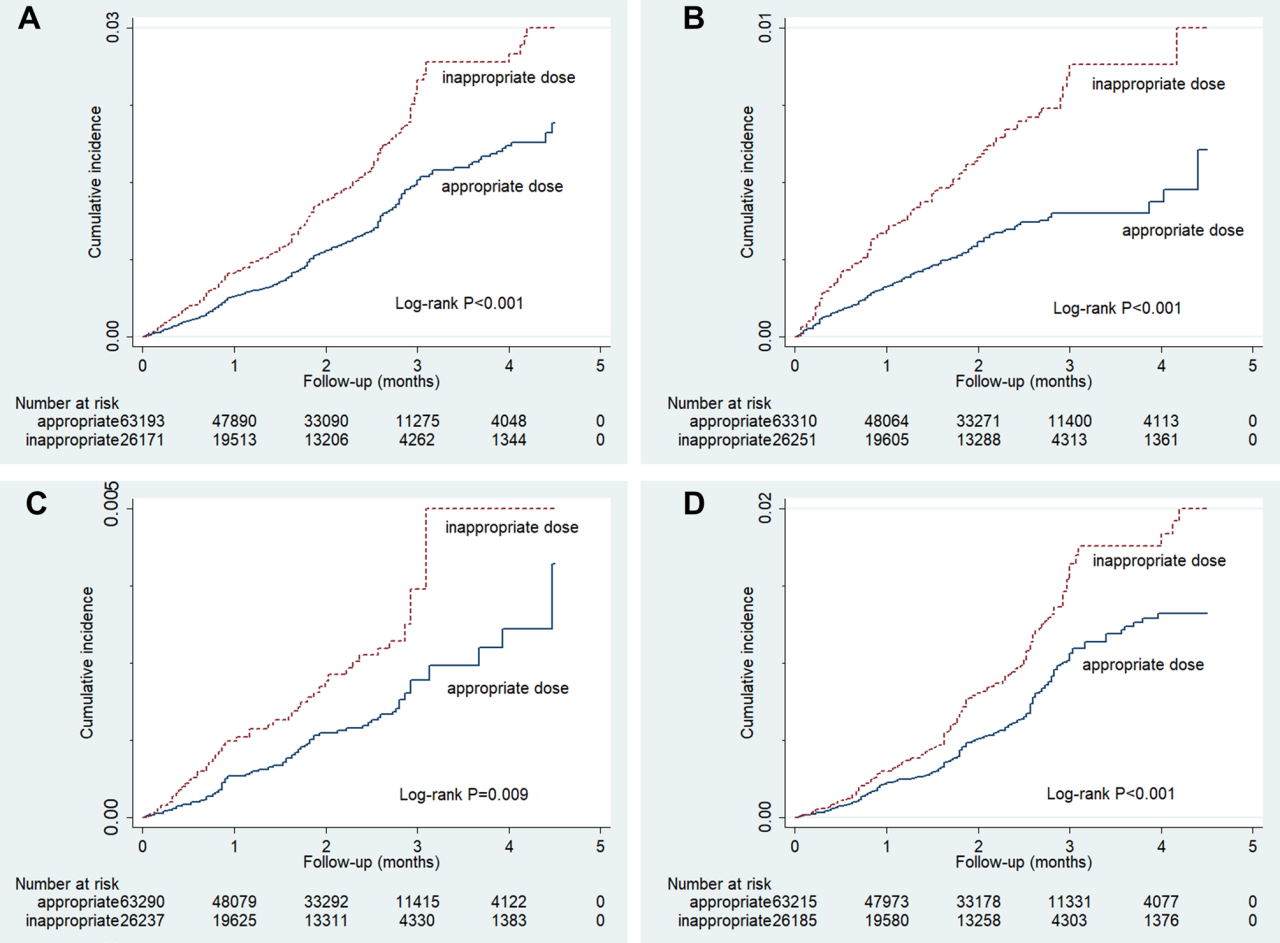
Kaplan–Meier cumulative incidence curve for (**A**) composite of all hypoglycemia, (**B**) severe hypoglycemia, (**C**) moderate hypoglycemia, and (**D**) mild hypoglycemia in patients receiving glucose-lowering drugs with eGFR < 50 mL/min/1.73 m^2^.

Table 2 presents the results of hypoglycemia risk associated with inappropriate and appropriate dosing. The incidence rate of the composite outcome of all hypoglycemia events was 76.71 and 48.51 events per 10,000 person-months in the inappropriate and appropriate dosing groups, respectively. The results of the Kaplan–Meier analysis showed that inappropriate dosing was associated with a higher cumulative incidence of hypoglycemia (log-rank *P* < 0.001 for severe, mild, and composite of all hypoglycemia, *P* = 0.009 for moderate hypoglycemia; Fig. 1). After adjusting for potential confounders, inappropriate dosing of glucose-lowering drugs was found to lead to an increased risk of the composite of all hypoglycemia (HR 1.52, 95% CI 1.31, 1.78). In addition, the results were consistent with regard to severe hypoglycemia (HR 1.87, 95% CI 1.40, 2.49), moderate hypoglycemia (HR 1.71, 95% CI 1.20, 2.43), or mild hypoglycemia (HR 1.46, 95% CI 1.21, 1.76).

**Table 2:** Risk of hypoglycemia associated with inappropriate and appropriate dosing.

Classification of hypoglycemia	Inappropriate dosing group			Appropriate dosing group			Unadjusted HR (95% CI)	Adjusted^a^ HR (95% CI)
	Events	Duration (person-month)	Incidence rate (events per 10,000 person-month)	Events	Duration (person-month)	Incidence rate (events per 10,000 person-month)		
Composite of all	417	54,359	76.71	651	134,207	48.51	1.58 (1.36, 1.83)	1.52 (1.31, 1.78)
Severe	97	54,658	17.75	120	134,801	8.90	1.98 (1.51, 2.61)	1.87 (1.40, 2.49)
Moderate	62	54,735	11.33	94	134,861	6.97	1.62 (1.16, 2.26)	1.71 (1.20, 2.43)
Mild	280	54,563	51.32	450	134,472	33.46	1.53 (1.28, 1.83)	1.46 (1.21, 1.76)

^a^Adjusted for patient age, sex, number of previous severe hypoglycemic events within the previous year, number of concurrent use of glucose-lowering drugs, use of insulin, use of sulfonylurea, Charlson Comorbidity Index, and HbA_1C_.

Supplementary Table 4 presents the incidence rates of the composite of all hypoglycemia by drug classifications. The incidence rates of sulfonylurea and acarbose were higher than those of the other classes of drugs.

### Subgroup and sensitivity analyses

**Figure 2:**
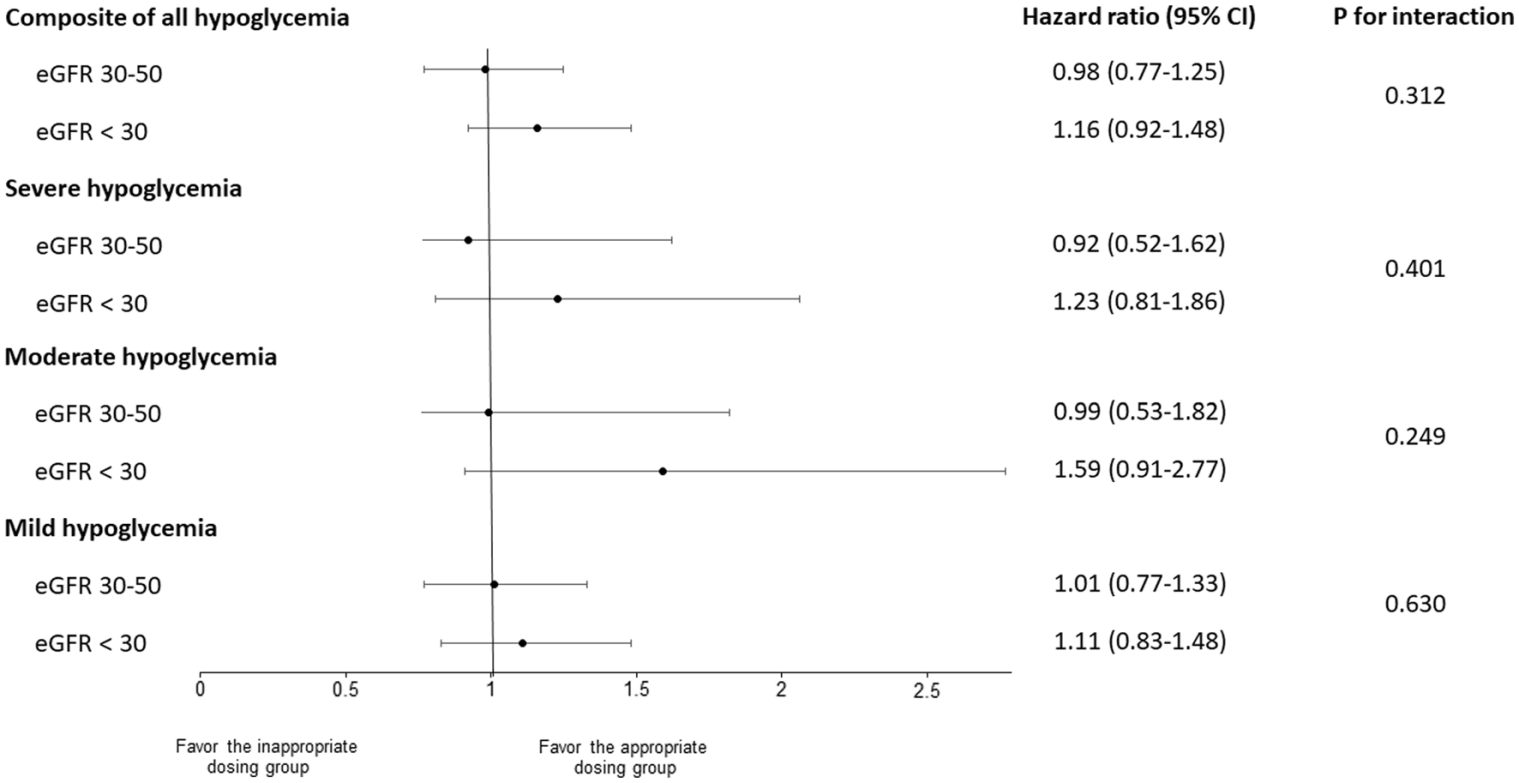
Risk of hypoglycemia in patients receiving glucose-lowering drugs with eGFR < 50 mL/min/1.73 m^2^ stratified by eGFR using marginal Cox proportional hazards regression analysis while controlling for baseline covariates. All analyses were adjusted for patient age, sex, number of severe hypoglycemic events within the previous year, number of concurrent use of glucose-lowering drugs, use of insulin, Charlson Comorbidity Index, and HbA_1C_. eGFR, estimated glomerular filtration rate (estimated using the body surface area modified Cockcroft–Gault formula).

Among patients with an eGFR < 30 mL/min/1.73 m^2^, the inappropriate dosing group had an increased but not significant risk of the composite of all hypoglycemia (HR 1.16, 95% CI 0.92, 1.48) as compared with the appropriate dosing group, as well as similar results for other types of hypoglycemia (Fig. 2; Supplementary Table 5). An eGFR < 30 mL/min/1.73 m^2^ did not increase the effects of inappropriate dosing of glucose-lowering drugs on the hypoglycemia risk of all types hypoglycemia (all *P* for interaction > 0.05).

In the sensitivity analysis (Supplementary TableS 6, 7, 8), HRs for inappropriate use remained significantly higher compared to appropriate use after excluding visits with insulin prescriptions, excluding dialysis patients, or using the CKD-EPI equation to calculate GFR.

## Discussion

In our observational cohort study, we found that inappropriate dosing of glucose-lowering drugs was associated with a 52% increase in the risk of the composite of all hypoglycemia in patients with CKD. To our knowledge, this study is the first to examine the associations between hypoglycemia based on the appropriateness of dosing with various glucose-lowering drugs.

We observed that inappropriate renal doses of antidiabetic drugs were prescribed in 29.3% of outpatient visits in the study population. This finding is similar to the results of previous studies that included only metformin and DPP-4 inhibitors^13,15,16^. Our study also included various glucose-lowering drugs that require renal adjustment for clinical use, such as metformin, sulfonylureas, DPP-4 inhibitors, sodium–glucose cotransporter-2 inhibitors, and acarbose. We conducted a more comprehensive analysis of the incidence of inappropriate renal dosing of general glucose-lowering drugs. With the introduction of various new glucose-lowering drugs, there is an increasing selection of drugs available for individualized treatment. Thus, it is important to note that the dose of some medications must to be adjusted according to patient renal function.

Our results indicated that incidence rates of all hypoglycemia for 48.51 and 76.71 events per 10,000 person-months (equal to 5.82 and 9.20 events per 100 person-years) in the appropriate and inappropriate dosing groups, respectively. Similar to the results reported in previous studies, the incidence rate of hypoglycemia among CKD patients receiving glucose-lowering drugs ranged from 2.35 to 10.16 events per 100 person-years^28,29^. In contrast, we noted a large difference from the findings of Hong et al., in that the incidence of severe hypoglycemia was 10.24 and 12.54 events per 1000 person-days (equal to 374 and 458 events per 100 person-years) in the appropriate dosing group and inappropriate dosing group, respectively^13^. However, comparing findings across studies is difficult because of differences in study designs, such as patient inclusion, clinical setting, and data collection. We believe that our study adds to the literature by reporting the incidence of hypoglycemia in patients with CKD who received inappropriate or appropriate dosing of glucose-lowering drugs.

The most common and important safety issue in patients receiving glucose-lowering drugs is hypoglycemia. In addition, patients with lower kidney function are at increased risk of hypoglycemia^29–31^. In patients with CKD, hypoglycemia is well established as increasing the risk of stroke, coronary heart disease, congestive heart failure, and even mortality^32–34^. Even mild hypoglycemia might be associated with cardiovascular disease in patients with CKD^35^. In our study, we found that the inappropriate renal dosing of glucose-lowering drugs was associated with a significantly increased risk of hypoglycemia in patients with CKD. Although we did not further analyze the direct association between hypoglycemia and the resulting outcomes in this study, we can still speculate that hypoglycemia may result in an adverse prognosis for patients.

In the subgroup analysis, the risk of hypoglycemia among patients with poorer renal function was not increased from the effect of inappropriate dosing of glucose-lowering drugs. Previous studies have shown that a reduced eGFR is associated with an increased risk of hypoglycemia^28,29^. The presence of impaired renal function can potentially influence the pharmacokinetics of glucose-lowering drugs, further decreasing in the drug clearance and increasing in plasma exposure, resulting in exposing patients to a higher risk of hypoglycemia^17^. However, our results might imply that the effects of inappropriate dosing may be greater than those from the reduced renal function. Thus, optimizing drug selection and administering the appropriate dose is critical, even in patients with moderate CKD.

The results of our study imply that in order to minimize harm in patients with CKD, special safeguards are required to reduce the risk of inappropriate drug dosing. The implementation of guided drug dosing, such as the adoption of a clinical decision support system (CDSS) and pharmacist-based interventions, could be considered. A CDSS integrated with computerized physician order entry systems for drug dosing has been shown to reduce prescribing errors and improve the overall quality of medicine use in patients with renal insufficiency^36–41^. In addition, the service provided by clinical pharmacists for patients with CKD can optimize drug treatment, reduce drug costs, and prevent adverse drug events^42–44^. The application of effective strategies for reducing inappropriate drug use is a priority for clinical caregivers at all times.

This is the first observational study examining the association between inappropriate renal dosing of various glucose-lowering drugs and episodes of hypoglycemia. The strength of our study is the inclusion of almost all glucose-lowering drug classes for which the dose must be adjusted in clinical use according to renal function. Furthermore, the present results were consistent across sensitivity analyzes.

Our study also has several limitations. First, because of the study’s retrospective observational study design, we were not able to access all residual confounders, such as lifestyle, other medical care, social support, and socioeconomic status. Second, the study data was obtained from a single hospital. Hypoglycemia data was not available for patients with hypoglycemia who presented to another hospital for assistance or who managed their disease on their own. However, this situation occurred in both groups. Therefore, the misclassification was nondifferential and might not have affected our results. Furthermore, our patients were older and more veterans. The lifestyle and awareness of hypoglycemia of our patients may be different from those of other groups. It remains unclear whether our results can be extrapolated to general patients. Third, in our study design, we integrated and analyzed all glucose-lowering drugs together for each outpatient visit and were unable to perform a follow-up analysis for a single type of glucose-lowering drug. However, to reduce this impact, we adjusted the number of glucose-lowering drugs in the outpatient visit. Fourth, we did not consider the effects of the drug–drug interaction on the risk of hypoglycemia. Finally, the patient’s medication adherence was unknown because the data on this factor were not available.

## Conclusion

The results of this study show that inappropriate dosing of glucose-lowering drugs in patients with CKD is common. Inappropriate dosing with glucose-lowering drugs was associated with a higher risk of hypoglycemia as compared with an appropriate dose of glucose-lowering drugs.

## Supplementary Information

Supplementary Information.

## Data Availability

The datasets generated and analyzed during the current study are not publicly available due the regulations of the Institutional Review Board of Taipei Veterans General Hospital. The data set is available on request from the corresponding author, or IRB of TVGH (email: irbopinion@vghtpe.gov.tw). The use of data is limited to research only.
