# Supplementary material for: Hypoglycemia risk with inappropriate dosing of glucose-lowering drugs in patients with chronic kidney disease: a retrospective cohort study
Source: Sci Rep. 2023 Apr 19;13:6373. doi: 10.1038/s41598-023-33542-z (PMC10115797; doi:10.1038/s41598-023-33542-z)
Supplement: Supplementary file 1 — Supplementary Information. [file 41598_2023_33542_MOESM1_ESM.docx]

**Hypoglycemia Risk with Inappropriate Dosing of Glucose-Lowering Drugs in Patients with Chronic Kidney Disease: A Retrospective Cohort Study**

Yun-Jhe Li, MSc; Yuh-Lih Chang, PhD; Yueh-Ching Chou, PhD; Chia-Chen Hsu, MSc

**Supplementary**

**
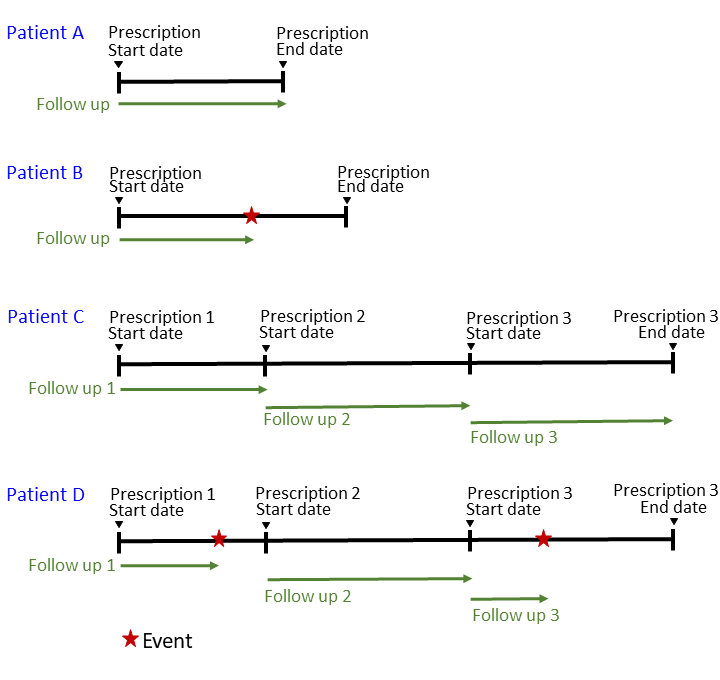
**

Supplementary Figure 1. Illustration of potential patterns of follow-up

***Patient A*** *had a visit with any of the target drugs. They were followed from the first date the target drugs were prescribed until the last date of prescription, without hypoglycemia events.* ***Patient B*** *had a visit with any of the target drugs. They were followed from the first date the target drugs were prescribed until the events occurred.* ***Patient C*** *had several visits with any of the target drugs. The first follow-up period was from the first date of prescription 1 to the first date of the next prescription (prescription 2). The second follow-up period was from the first date of prescription 2 to the first date of prescription 3. And so on.* ***Patient D*** *had several visits with any of the target drugs. The first follow-up period was from the first date of prescription 1 to the date the event occurred. The second follow-up period was from the first date of prescription 2 to the first date of prescription 3. The third follow-up period was from the first date of prescription 3 to the date the event occurred. And so on.*

Supplementary Table 1. Target drugs with renal dosing recommendations

| Drug | Renal function | Dosing recommendation |
| --- | --- | --- |
| Acarbose | eGFR < 25 | Contraindicated |
| Alogliptin | eGFR 30–50 | 12.5 mg once daily |
|  | eGFR < 30, HD | 6.25 mg once daily |
| Dapagliflozin | eGFR 30–45 | Not recommended |
|  | eGFR< 30, ESRD, HD | Contraindicated |
| Empagliflozin | eGFR< 30, ESRD, dialysis | Contraindicated |
| Gliclazide | eGFR < 30 | Contraindicated |
| Glimepiride | eGFR < 30 | Contraindicated |
| Metformin | eGFR 30–45 | Maximum 1 g per day |
|  | eGFR < 30 | Contraindicated |
| Saxagliptin | eGFR < 50, HD | 2.5 mg once daily |
| Sitagliptin | eGFR 30–45 | 50 mg once daily |
|  | eGFR < 30, HD, PD | 25 mg once daily |
| Vildagliptin | eGFR < 50, HD | 50 mg once daily |

eGFR, estimated glomerular filtration rate (mL/min/1.73 m^2^); ESRD, end-stage renal disease; HD, hemodialysis; PD, peritoneal dialysis.

Supplementary Table 2. Criteria for different severity of hypoglycemia

| Classification | Criteria |
| --- | --- |
| Severe hypoglycemia | Emergency visit due to hypoglycemia with blood glucose level < 70 mg/dL or any of the following ICD codes:  ICD-9 code: (x indicates any number)  250.3x, 250.8x, 251.0, 251.1, 251.2, 962.3  ICD-10 code:  E08.641, E08.649, E09.641, E09.649, E10.641, E10.649, E10.69, E11.641, E11.649, E11.69, E13.641, E13.649, E15, E16.0, E16.1, E16.2, T38.3X1A, T38.3X1D, T38.3X2A, T38.3X2D, T38.3X3A, T38.3X3D, T38.3X4A, T38.3X4D, T38.3X5A, T38.3X5D |
| Moderate hypoglycemia | Outpatient visit with blood glucose level < 54 mg/dL |
| Mild hypoglycemia | Outpatient visit with blood glucose level between 54 and 69 mg/dL |
| Composite of all hypoglycemia | Hypoglycemia with any severity of mentioned above |

Supplementary Table 3. Frequency of drug prescription according to the patient’s renal function

| Drugs | Total | eGFR (mL/min/1.73 m^2^) | | | |
| --- | --- | --- | --- | --- | --- |
|  |  | 30-50 | 15-29 | <15^a^ | dialysis |
| Acarbose | 18,666 | 12,018 | 5,723 | 836 | 89 |
| Alogliptin | 99 | 83 | 10 | 1 | 5 |
| Dapagliflozin | 225 | 199 | 26 | 0 | 0 |
| Empagliflozin | 2,010 | 1,806 | 202 | 2 | 0 |
| Gliclazide | 12,986 | 9,644 | 2,809 | 347 | 186 |
| Glimepiride | 23,132 | 17885 | 4,837 | 370 | 40 |
| Metformin | 52,769 | 44,962 | 7,658 | 173 | 3 |
| Saxagliptin | 4,969 | 3,549 | 997 | 238 | 185 |
| Sitagliptin | 16,218 | 12,089 | 3,447 | 474 | 208 |
| Vildagliptin | 9,240 | 5,036 | 2,769 | 1,025 | 410 |

^a^ Patients did not receiving dialysis

Supplementary Table 4. Incidence rates of the composite of all hypoglycemia by drug classifications

| Drug classifications | Inappropriate use | | |  | Appropriate use | | |
| --- | --- | --- | --- | --- | --- | --- | --- |
|  | Events | Duration (person-month) | Incidence rate (events per 10,000 person-month) |  | Events | Duration (person-month) | Incidence rate (events per 10,000 person-month) |
| Metformin | 142 | 25,555 | 55.57 |  | 369 | 89,823 | 41.08 |
| DPP-4 inhibitors | 216 | 34,027 | 63.48 |  | 174 | 31,105 | 55.94 |
| Sulfonylureas | 258 | 24,944 | 103.43 |  | 320 | 50,821 | 62.97 |
| Acarbose | 112 | 12,541 | 89.31 |  | 154 | 26,181 | 58.82 |
| SGLT-2 inhibitors | 1 | 482 | 20.75 |  | 21 | 3,993 | 41.08 |

Supplementary Table 5. Risk of hypoglycemia associated with inappropriate and appropriate dosing, subgroup analysis stratified by eGFR

|  | Inappropriate dosing group | | |  | Appropriate dosing group | | |  | Adjusted^a^ HR (95% CI) |  | *P* for interaction |
| --- | --- | --- | --- | --- | --- | --- | --- | --- | --- | --- | --- |
|  | Events | Duration (person-month) | Incidence rate (events per 10,000 person-month) |  | Events | Duration (person-month) | Incidence rate (events per 10,000 person-month) |  |  |  |  |
| **Composite of all hypoglycemia** |  |  |  |  |  |  |  |  |  |  | 0.312 |
| eGFR 30–50 | 103 | 24,358 | 42.29 |  | 514 | 117,047 | 43.91 |  | 0.98 (0.77, 1.25) |  |  |
| eGFR < 30 | 314 | 30,001 | 104.66 |  | 137 | 17,160 | 79.84 |  | 1.16 (0.92, 1.48) |  |  |
| **Severe hypoglycemia** |  |  |  |  |  |  |  |  |  |  | 0.401 |
| eGFR 30–50 | 18 | 24,450 | 7.36 |  | 85 | 117,533 | 7.23 |  | 0.92 (0.52, 1.62) |  |  |
| eGFR < 30 | 79 | 30,207 | 26.15 |  | 35 | 17,268 | 20.27 |  | 1.23 (0.81, 1.86) |  |  |
| **Moderate hypoglycemia** |  |  |  |  |  |  |  |  |  |  | 0.249 |
| eGFR 30–50 | 13 | 24,459 | 5.32 |  | 75 | 117,565 | 6.38 |  | 0.99 (0.53, 1.82) |  |  |
| eGFR < 30 | 49 | 30,276 | 16.18 |  | 19 | 17,296 | 10.99 |  | 1.59 (0.91, 2.77) |  |  |
| **Mild hypoglycemia** |  |  |  |  |  |  |  |  |  |  | 0.630 |
| eGFR 30–50 | 76 | 24,398 | 31.15 |  | 363 | 117,239 | 30.96 |  | 1.01 (0.77, 1.33) |  |  |
| eGFR < 30 | 204 | 30,165 | 67.63 |  | 87 | 17,233 | 50.49 |  | 1.11 (0.83, 1.48) |  |  |

^a^ Adjusted for patient age, sex, number of previous of severe hypoglycemic events within the previous year, number of concurrent use of glucose-lowering drugs, use of insulin, use of sulfonylurea, Charlson Comorbidity Index, and HbA_1C_.

Supplementary Table 6. Sensitivity analysis to exclude visits with insulin prescriptions

| Classification of hypoglycemia | Inappropriate dosing group  n=21,778 | | |  | Appropriate dosing group  n=52,233 | | | Unadjusted HR (95% CI) | Adjusted^a^  HR (95% CI) |
| --- | --- | --- | --- | --- | --- | --- | --- | --- | --- |
|  | Events | Duration (person-month) | Incidence rate (events per 10,000 person-month) |  | Events | Duration (person-month) | Incidence rate (events per 10,000 person-month) |  |  |
| Composite of all | 273 | 45,515 | 59.98 |  | 375 | 111,882 | 33.52 | 1.79  (1.48, 2.15) | 1.67  (1.37, 2.03) |
| Severe | 69 | 45,700 | 15.10 |  | 84 | 112,206 | 7.49 | 2.00  (1.45, 2.77) | 1.75  (1.23, 2.49) |
| Moderate | 37 | 45,759 | 8.09 |  | 44 | 112,272 | 3.92 | 2.07  (1.33, 3.23) | 2.10  (1.30, 3.37) |
| Mild | 182 | 45,648 | 39.87 |  | 257 | 112,038 | 22.94 | 1.74  (1.38, 2.18) | 1.62  (1.28, 2.05) |

^a^ Adjusted for patient age, sex, number of previous severe hypoglycemic events within the previous year, number of concurrent use of glucose-lowering drugs, use of sulfonylurea, Charlson Comorbidity Index, and HbA_1C_

Supplementary Table 7. Sensitivity analysis to exclude visits of patients with dialysis

| Classification of hypoglycemia | Inappropriate dosing group | | |  | Appropriate dosing group | | | Unadjusted HR (95% CI) | Adjusted^a^  HR (95% CI) |
| --- | --- | --- | --- | --- | --- | --- | --- | --- | --- |
|  | Events | Duration (person-month) | Incidence rate (events per 10,000 person-month) |  | Events | Duration (person-month) | Incidence rate (events per 10,000 person-month) |  |  |
| Composite of all | 407 | 53,823 | 75.62 |  | 638 | 133,471 | 47.80 | 1.58  (1.36, 1.83) | 1.52  (1.30, 1.78) |
| Severe | 96 | 54,115 | 17.74 |  | 115 | 134,058 | 8.58 | 2.06  (1.56, 2.72) | 1.94  (1.44, 2.60) |
| Moderate | 57 | 54,195 | 10.52 |  | 92 | 134,114 | 6.86 | 1.53  (1.09, 2.16) | 1.59  (1.10, 2.30) |
| Mild | 276 | 54,022 | 51.09 |  | 442 | 133,730 | 33.05 | 1.54  (1.29, 1.85) | 1.47  (1.21, 1.78) |

^a^ Adjusted for patient age, sex, number of previous severe hypoglycemic events within the previous year, number of concurrent use of glucose-lowering drugs, use of sulfonylurea, use of insulin, Charlson Comorbidity Index, and HbA_1C_

Supplementary Table 8. Sensitivity analysis using CKD-EPI equation to estimate GFR

| Classification of hypoglycemia | Inappropriate dosing group  n=14,758 | | |  | Appropriate dosing group  n=42,055 | | | Unadjusted HR (95% CI) | Adjusted^a^  HR (95% CI) |
| --- | --- | --- | --- | --- | --- | --- | --- | --- | --- |
|  | Events | Duration (person-month) | Incidence rate (events per 10,000 person-month) |  | Events | Duration (person-month) | Incidence rate (events per 10,000 person-month) |  |  |
| Composite of all | 312 | 29,230 | 106.74 |  | 527 | 89,867 | 58.64 | 1.80  (1.51, 2.16) | 1.65  (1.38, 1.98) |
| Severe | 66 | 29,450 | 22.41 |  | 98 | 90,366 | 10.84 | 2.05  (1.48, 2.85) | 1.92  (1.36, 2.70) |
| Moderate | 49 | 29,511 | 16.60 |  | 67 | 90,413 | 7.41 | 2.19  (1.50, 3.20) | 2.20  (1.49, 3.24) |
| Mild | 212 | 29,363 | 72.20 |  | 380 | 90,086 | 42.18 | 1.70  (1.37, 2.11) | 1.51  (1.22, 1.88) |
